# Obesity: physiologic changes and implications for preoperative management

**DOI:** 10.1186/s12871-015-0079-8

**Published:** 2015-07-04

**Authors:** Vilma E. Ortiz, Jean Kwo

**Affiliations:** Department of Anesthesia, Critical Care & Pain Medicine, Associate Anesthetist, Massachusetts General Hospital, 55 Fruit Street, Boston, MA 02114 USA

**Keywords:** Obesity, Morbid obesity, Preoperative evaluation, Metabolic syndrome, Obstructive sleep apnea (OSA), Bariatric, Body mass index, Insulin resistance

## Abstract

The proportion of patients defined as obese continues to grow in many westernized nations, particularly the United States (USA). This trend has shifted the perioperative management of obese patients into the realm of routine care. As obese patients present for all types of procedures, it is crucial for anesthesiologists, surgeons, internists, and perioperative health care providers alike to have a firm understanding of their altered multi-organ physiology in order to safely prepare the obese patient for an operation. A careful preoperative evaluation may also serve to identify risk factors for postoperative adverse events. Subsequently, preoperative measures may be implemented to mitigate these complications. In this manuscript we address the major considerations for the preoperative evaluation of the severely obese patient.

## Introduction

*“Thou seest I have more flesh than another man, and therefore more frailty” W. Shakespeare (1564–1616), Henry IV.*

A condition known since antiquity, obesity has ‘grown’ from being the exception to a global public health challenge. A recent study reveals that between 1980 and 2013 the worldwide rates of overweight and obesity have increased by 28 % in adults and 47 % in children. As of 2013, 2.1 billion are overweight and obese compared to 857 million in 1980. The United States is home to approximately 13 % of the world’s 671 million obese individuals [1]. The comorbidities associated with excess weight result in a health burden that has a significant economic impact [2].

Obesity is associated with conditions such as type 2 diabetes (T2DM), chronic kidney disease [3, 4], depression [5], stroke [6] and coronary artery disease (CAD) [7]. These comorbidities, in addition to the type and invasiveness of the surgical procedure, are correlated with the incidence and severity of postoperative complications [8, 9]. Excess weight is also associated with conditions that in and of themselves increase the likelihood of needing surgery and anesthesia: malignancies – notably, cervical, endometrial, colorectal and gallbladder cancer [9]; osteoarthritis [10]; back pain [11]; stress incontinence [12] and gallstones [13]. These conditions are a result of both physiologic changes as well as inflammatory changes associated with obesity.

In this manuscript, we aim to review the major obesity-related co-morbidities and their impact on the preoperative evaluation. Wherever data and/or expert opinion exist, we provide guidance for perioperative management.

## Review

### Definition of obesity

The diagnosis of obesity is often based on body mass index (BMI) calculated as weight in kilograms divided by height in meters, squared. A BMI of 18.5 to 24.9 is considered normal, a BMI of 25–29.9 overweight and a BMI > 30 is considered obese. Obesity is further categorized into Class I (BMI 30 – 34.9), Class II (BMI 35 – 39.9) and Class III (BMI > 40) [14]. BMI is a global measure of body mass encompassing both adipose tissue and lean mass. It accounts for neither the proportion of each tissue nor the regional distribution of adipose tissue, factors which can have important implications for the clinical assessment of patients. Independent of total weight, excess adiposity in the central area (visceral or intra-abdominal) is associated with higher insulin resistance and risk of atherosclerotic heart disease than a more peripheral distribution of fat (gluteofemoral) [15, 16]. Consequently, indirect measures of central fat distribution, such as waist circumference or waist-to-hip ratio (WHR), may be better markers of obesity-related comorbidities such as CAD [7] and dyslipidemia [17].

## Major changes associated with obesity

### Inflammatory changes

Beyond its role in energy balance and the mechanical load it imposes, adipose tissue impacts our bodies through its endocrine properties. Excess weight results when calories consumed exceed calories spent. The positive energy balance eventually leads to adipose tissue hypertrophy, macrophage recruitment and to complex adaptive changes in the adipocytes, their supporting tissue, blood supply and immunological milieu. Over time, adipocyte cell death and chronic tissue hypoxia occur as the supply of nutrients and oxygen trails behind the demands of the hypertrophic cells [18]. Among the obesity-induced changes in adipose tissue activity is the altered secretion of cell-signaling proteins known as adipokines and the increased production of inflammatory markers such as TNF-α and IL-6 by resident macrophages [19, 20]. These mediators interact with the sympathetic nervous system, the renin-angiotensin-aldosterone system and individual organs -such as the pancreas and liver- to effect the alterations in physiology that accompany obesity [19, 21].

#### Metabolic syndrome

The chronic low-grade state of inflammation and immune system activation that typifies obesity promotes insulin resistance and the development of T2DM [22]. The metabolic syndrome is a group of conditions more likely to occur together than randomly alone and increase the risk of cardiovascular disease and T2DM. A recent definition includes: elevated waist circumference (value determined by individual populations) plus any two of the following: elevated triglycerides (≥150 mg/dl), reduced HDL-C (≤40 mg/dl in males, ≤50 mg/dl in females), hypertension (systolic ≥ 130 and/or diastolic ≥ 85 mmHg) and elevated fasting glucose (≥100 mg/dl) [23].

## Physiologic changes

### Cardiovascular

Changes in the cardiac system occur as a consequence of the cardiovascular adaptation to excess body mass and increased metabolic demands [24]. Approximately 31 % of individuals with extreme obesity, particularly if long-standing, will develop structural and functional changes that lead to obesity cardiomyopathy [25, 26]. Excess body mass requires an increase in intravascular blood volume as well as an increase in cardiac output (mostly from an increase in stroke volume). Over time, the increase in stroke volume leads to an increase in left ventricular load, dilation and compensatory left ventricular hypertrophy, a known precursor of heart failure [27]. Mechanical impairment also results from structural remodeling driven by prolonged exposure to factors that exert direct cardiotoxic effects (e.g. insulin resistance, steatosis, neurohumoral over-activation) as well as the repeated cycles of nocturnal hypoxia and hypercarbia associated with intermittent airway obstruction characteristic of obstructive sleep apnea. These eventually culminate in pulmonary hypertension and biventricular dysfunction [28, 29].

#### Coronary artery disease

Risk factors for coronary artery disease (CAD) in patients with obesity include: T2DM, hypertension, dyslipidemia, heightened inflammation, and a prothrombotic state. In a meta-analysis of 19,388 bariatric surgery patients, 7 % had a history of CAD [8]. Unfortunately, symptoms associated with coronary ischemia, such as dyspnea on exertion and chest pain, occur commonly in obese patients and can be nonspecific [30].

The American Association of Clinical Endocrinologists, the Obesity Society, and the American Society of Metabolic and Bariatric Surgery (AACE-TOS-ASMBS) and the American College of Cardiologists and the American Heart Association (ACC-AHA) have both published guidelines for the perioperative evaluation of the bariatric patient [31]. A preoperative electrocardiogram (ECG) should be obtained if cardiac disease is suspected (i.e. patient has a risk factor for CAD or has known stable cardiovascular disease). Obtaining a routine ECG in all-comers can lead to unnecessary tests prompted by false positive results. In a study of bariatric surgery patients, 62 % had ECG abnormalities which, when evaluated by a cardiologist, did not constitute a contraindication to bariatric surgery [32]. Signs of right ventricular hypertrophy (right axis deviation, right bundle branch block) on ECG may indicate pulmonary hypertension, a significant risk factor for postoperative complications [33]. A new left bundle branch block may signal occult CAD. Both findings should prompt additional investigation.

The need for further cardiac evaluation depends on an in-depth analysis of the patient’s risk factors, cardiac risk associated with the planned procedure and the patient’s functional status. Older studies reported cardiac complications in 1 % to 1.4 % of patients undergoing open bariatric surgical procedures [34]. More recently, less invasive procedures such as laparoscopic Roux-en-Y gastric bypass and laparoscopic gastric banding have resulted in a lower risk of cardiac complications (<0.5 %) [35]. This underscores the pivotal role of procedural risk factors - in addition to patient risk factors - in assessing cardiac risk. The Revised Cardiac Risk Index [36] and the American College of Surgeons NSQIP Risk Calculator (access at: http://www.surgicalriskcalculator.com) [37] are validated tools to help estimate perioperative risk. Guidelines published by the ACC-AHA [38] stipulate no further cardiac testing if the risk of a major adverse cardiac event (MACE) is < 1 %. If the MACE risk is ≥ 1 %, functional status should be ascertained.

Functional status can be inferred from an individual’s ability to perform activities of daily living. The ability to perform > 4 metabolic equivalents (METS) of activity is associated with a low risk of perioperative cardiac events [39]. Patients undergoing bariatric surgery with a functional capacity of < 4.5 METs as measured by exercise testing had a 16.7 % complication rate (defined as death, unstable angina, myocardial infarction, venous thromboembolism (VTE), renal failure, or stroke). In contrast, the complication rate in patients with a functional capacity > 4.5 METs was 2.8 % [40]. Importantly, the assessment of functional status is often difficult because of limited mobility in patients with severe obesity [30]. For the patient whose functional capacity is < 4 METS and whose risk of MACE is high (≥1 %), stress testing represents a useful modality to determine ischemic potential. Potential challenges include a patient’s inability to exercise, lack of equipment to support excess weight, and inability to obtain high-quality echocardiographic images due to body habitus. If decreased cardiac function is uncovered, cardiomyopathy secondary to obesity, diabetes, or long-standing ischemia ought to be considered. Cardiomyopathy predisposes to heart failure, which entails higher risks of operative mortality and hospital readmission [41].

#### Hypertension

Hypertension, traditionally defined as a systolic blood pressure ≥ 140 mmHg or a diastolic blood pressure of ≥ 90 mmHg [42], is an independent risk factor for the development of CAD, stroke and structural end-organ injury [43]. A direct relationship between excess weight and elevated blood pressure has been demonstrated in several population-based studies [44–46].

The etiology of hypertension in the setting of obesity and the metabolic syndrome is multifactorial, resulting from the interaction between genetic factors, insulin resistance, sodium retention, activation of the sympathetic nervous system as well as the activation of the renin-angiotensin-aldosterone axis. No consensus currently exists on the optimal antihypertensive drug regimen in the obese. However, angiotensin-converting-enzyme (ACE) inhibitors and angiotensin receptor blockers (ARBs) are favored by some investigators because of their ability to increase insulin sensitivity, thus diminishing the risk of diabetes mellitus [47]. To enhance its effectiveness, treatment of obesity-related hypertension must include emphasis on weight management through dietary and behavioral modifications.

#### Arrhythmias

Arrhythmias in the obese may be precipitated by hypoxemia, left atrial and ventricular enlargement, electrolyte disturbances from diuretic therapy, increase in plasma catecholamines and hypercarbia. A robust association between obesity and atrial fibrillation (AF) [48] was demonstrated in a cardiothoracic study where patients with a BMI > 40 had a 2.3 fold increased risk of postoperative AF compared to a 1.2 fold increased risk in those with a BMI between 25 and 30 [49]. Despite this association, it is unclear whether AF confers a greater risk of adverse perioperative cardiac events. Guidelines published by the ACC/AHA note that “a paucity of studies that address surgical risk conferred by arrhythmias limits the ability to provide specific recommendations” [38]. However, a retrospective administrative database study of 38,047 patients showed that a prior admission for AF was associated with a higher perioperative mortality rate than CAD [50]. Preoperative evaluation of the patient with a history of AF involves an assessment of rate and/or rhythm control as well as a review of medications, particularly anticoagulants [38]. Patients with AF can be managed with either heart rate control or conversion to normal sinus rhythm as both AF and normal sinus rhythm have comparable rates of mortality and stroke in this population [51]. Current guidelines recommend a target heart rate of less than 80 beats per minute, though a “lenient” rate-control strategy (resting heart rate < 110 beats per minute) may be considered in asymptomatic patients with preserved LV function [52].

The CHA2DS2-VASc score (Table 1) should be used for risk stratification of thromboembolic risk in patients with AF [52]. Patients with a CHA2DS2-VASc score greater than 2 should receive anticoagulation therapy. Perioperatively, the decision of whether or not to continue anticoagulation and the appropriate regimen for each patient should balance the risk of bleeding with the risk of stroke if therapy is discontinued.

**Table 1:** CHA2DS2-VASc Score

Parameter	Score
**C**ongestive Heart Failure	1
**H**ypertension	1
**A**ge ≥ 75	2
**A**ge 65-74	1
**D**iabetes Mellitus	1
**S**troke/Transient ischemic attack	2
**V**ascular disease (prior MI, peripheral arterial disease, aortic plaque)	1
**S**ex **c**ategory (Female)	1

### Respiratory

Obesity’s impact on respiratory function (Table 2) is inversely related to BMI, with significant impairment observed once BMI exceeds 45 [53]. Consistent with restrictive physiology, the pattern of distribution of excess weight - central vs. peripheral - is more consequential than BMI *per se*. For further discussion, the reader is referred to the BMC Anesthesiology article by Fernandez-Bustamante *et al.* [54].

**Table 2:** Respiratory changes with obesity [119–124]

Parameter	Obesity-Related Change
Work of breathing (WOB)	Increased
Functional residual capacity (FRC)	Decreased
Expiratory reserve volume (ERV)	Decreased
Total lung capacity (TLC)	Unchanged, though decreased in severe obesity
Vital capacity (VC)	Decreased
Forced expiratory volume in 1 s (FEV_1_)	Unchanged, though decreased in severe obesity
Forced vital capacity (FVC)	Unchanged, though decreased in severe obesity
FEV_1_/FVC	Unchanged, though decreased in severe obesity
Diffusing capacity of the lung for carbon monoxide (DLCO)	Unchanged in simple obesity

#### Obstructive sleep apnea

Magnetic resonance imaging studies confirm that pharyngeal structures (from the nasopharynx to the laryngopharynx) increase in size with deposition of adipose tissue [55]. Additionally, King *et al.* have documented a reduction in airway caliber (increase in airway resistance) with increasing weight [56]. These changes in pharyngeal shape are associated with impairment of pharyngeal dilator activity and an increased risk of airway collapse [57]. Although obstruction may occur at any point in the pharynx, it is most frequently observed in either the retropalatal and/or the retroglossal regions [57].

Obstructive sleep apnea (OSA), a sleep-related breathing disorder, is estimated to affect between 40 % and 90 % of obese individuals [57]. It is characterized by periodic reduction or cessation of breathing due to narrowing of the upper airways during sleep. Factors linking obesity and OSA include anatomical imbalance from excess upper airway fat deposition, changes in upper airway muscle tone [58, 59], as well as alterations in the control of ventilation [60]. Furthermore, OSA itself leads to changes that contribute to the development of obesity: decreased energy level, motivation, sleep fragmentation *etc.* While the majority of individuals with severe obesity are able to maintain eucapnia, a significant minority will develop obesity hypoventilation syndrome (OHS), characterized by alveolar hypoventilation (PaCO_2_ > 45 mmHg) unexplained by other disorders [61, 62].

OSA can negatively affect perioperative outcome. The Longitudinal Assessment of Bariatric Surgery (LABS) study found that a history of OSA was significantly associated with a composite endpoint of death, VTE, reintervention, or failure to be discharged by 30 days after surgery [63]. However, preoperative intervention may reverse this impact. Weingarten *et al.* did not find an association between OSA and postoperative respiratory, cardiac, or surgical complications in affected patients who were treated preoperatively with continuous positive airway pressure (CPAP) or bi-level positive airway pressure (biPAP) for several weeks to months and were monitored with pulse oximetry postoperatively [64].

As OSA is often undiagnosed, routine polysomnography (PSG) for patients undergoing bariatric surgery has been recommended [32, 65]. Though this test is the gold standard for diagnosis, it is costly and time-consuming. Furthermore, whether or not routine screening improves safety and outcomes is debatable. A study of 1,058,710 patients undergoing elective orthopedic, abdominal, prostate, and cardiovascular surgery found that sleep-disordered breathing (SDB) was not associated with a clinically significant increase in in-hospital mortality, length of stay or total charges [66]. However, patients with SDB were more likely to have cardiopulmonary complications such as AF, respiratory failure, emergency intubation, as well as non-invasive and mechanical ventilation.

A protocol for the evaluation of patients at risk for OSA is an integral component of the preoperative assessment of the obese [67]. Questions regarding snoring, apneic episodes, frequent arousals during sleep, morning headaches, and daytime somnolence should be explored. The physical examination should include an evaluation of the airway, neck circumference, tongue size and volume, and nasopharyngeal characteristics. Despite varying sensitivities and specificities, tools such as the STOP-Bang questionnaire [68], Epsworth Sleepness Scale [69] or the Berlin questionnaire [70] can facilitate the OSA screening process. The STOP-Bang questionnaire (Table 3) [68], developed specifically for use in surgical patients, has been validated in patients with a BMI > 30 [71]. In the obese, a STOP-Bang score of ≥ 3 has a sensitivity of 90.5 % for detecting OSA with a positive predictive value of 84.8 %. A score of ≥ 5 is associated with a sensitivity of 53 % and a specificity of 70.2 % for predicting moderate/severe OSA (defined as an apnea-hypopnea index [AHI] >15) and a sensitivity of 68.8 % and a specificity of 68.7 % for predicting severe OSA (AHI > 30).

**Table 3:** STOP-BANG questionnaire

**S**noring	Do you **Snore Loudly**?
**T**ired	Do you often feel **Tired, Fatigued, or Sleepy** during the daytime?
**O**bserved	Has anyone **Observed** you **Stop Breathing** or **Choking/Gasping** during your sleep?
**P**ressure	Do you have or are you being treated for **High Blood Pressure**?
**B**ody Mass Index	BMI **> 35 kg/m**^**2**^
**A**ge	Age **> 50 years**
**N**eck Circumference	Shirt collar **> 17 in/43 cm for males**
	Shirt collar **> 16 in/41 cm for females**
**G**ender	Gender = **male**

The STOP-Bang questionnaire is a screening tool for OSA. In obese patients, a score of 0–3 indicates a low risk of OSA, a score of 4–5, an intermediate risk of OSA, and a score of 6–8, a high risk of OSA [71]. Adapted from http://www.stopbang.ca/screen.php

**Table 4:** Major obesity-related conditions and pertinent studies

Organ System	Major issues	Pertinent studies
Cardiovascular		• ECG if cardiac disease is suspected
	Coronary artery disease	• Use validated tools to estimate risk of perioperative MACE
		• If risk of MACE ≥ 1 % and functional status is poor, consider stress testing
	Pulmonary hypertension	• Consider right ventricular hypertrophy, pulmonary hypertension if ECG shows right axis deviation, right bundle branch block
		• Echocardiogram to assess right and left ventricular function & morphology, valvular morphology, estimate pulmonary artery pressure
		• Right heart catherization
	Congestive heart failure	• Chest radiograph
		• Echocardiogram
Respiratory		
	Dyspnea	• Chest radiograph
	Asthma	• Pulmonary function testing not recommended for routine screening
	Obstructive sleep apnea	• Screen for OSA with history, physical exam, validated screening questionnaire
		• Consider polysomnogram
		• Consider initiating CPAP/biPAP preoperatively
	Hypoventilation syndrome	• Arterial blood gas
Gastrointestinal		
	GERD	• Upper endoscopy
		• 24-h pH monitoring
		• Esophageal manometry
		• Barium swallow (upper gastrointestinal series)
	NAFLD	• Liver function tests (LFTs)
		• Triglyceride level
		• Liver ultrasound if LFTs elevated or symptomatic biliary disease
	H. Pylori	• Stool antigen test
		• Urea breath test
		• Endoscopy – rapid urease test
Endocrine		
	Diabetes mellitus	• Measure Hgb A1c
		• Optimize glycemic control
Hematologic		
	VTE	• Assess VTE risk: degree of obesity, age, history of previous DVT or hypercoagulable state, history of malignancy, immobility
Psychologic		• Psychosocial-behavioral evaluation
	Depression / anxiety	• Identify patients at risk for suicide
	Binge eating disorder	
Nutritional		• Iron studies, B_12_, folate, 25-hydroxyvitamin D
		• Electrolytes, calcium, magnesium, phosphate levels

When clinical screening identifies a patient as potentially having OSA, the decision whether to manage him clinically preoperatively or to obtain sleep studies and initiate OSA treatment prior to surgery should take into account the severity of OSA (based on clinical indicators or sleep study results), the invasiveness of the planned procedure, and the estimated postoperative narcotic requirement [67]. A recent Cochrane review found no evidence that CPAP reduces postoperative mortality; however, it may offer benefit in preventing pneumonia, reducing atelectasis, and lowering the risk of reintubation [72]. While studying the incidence of serious postoperative complications (e.g. cardiac events) in OSA patients, Gupta *et al.* found a lower incidence in those treated with preoperative CPAP than in the untreated group [73]. In a recent trial, OSA patients treated with CPAP for a 12-week period showed a reduction in mean arterial pressure whereas those who received only nocturnal supplemental oxygen or education did not [74]. Despite insufficient data to conclusively establish the benefits of pre- and postoperative CPAP, the American Society of Anesthesiologists (ASA) [67] recommends considering the perioperative initiation of CPAP, particularly if OSA is severe. In patients already treated with CPAP or non-invasive positive pressure ventilation (NIPPV), this treatment should be continued into the postoperative period unless contraindicated [67].

#### Asthma

A meta-analysis involving 333,102 subjects demonstrated that obese individuals (BMI > 30) were nearly twice as likely to have asthma as individuals with a BMI < 25 [75]. Predisposing factors included: decreased airway caliber due to decreased lung volumes; altered airway smooth muscle contractility leading to increased airway responsiveness and the chronic, low-grade inflammatory state associated with obesity [76]. Obese patients are more likely to have severe asthma and to respond poorly to inhaled corticosteroids and long-acting beta agonists. Weight loss is associated with improved asthma control [76].

#### Pulmonary hypertension

Obese patients have multiple risk factors for developing pulmonary hypertension (PH) such as OSA, OHS, left heart dysfunction, and chronic pulmonary thromboembolism. Patients with PH are at increased risk for morbidity and mortality with anesthesia and surgery. Kaw *et al.* reported that 26 % of patients with PH (defined as a mean pulmonary artery pressure of > 25 mmHg) experienced postoperative morbidity or mortality as compared to a 2.6 % complication rate in patients without PH. Compared to controls, patients with PH had a significantly higher risk of developing congestive heart failure, respiratory failure, hemodynamic instability and sepsis. Factors associated with increased risk of complications included poor functional status (New York Heart Association functional class ≥ II), history of pulmonary embolism, OSA, and surgical complexity [77]. Other risk factors included longer time under anesthesia (>3 h) and intraoperative vasopressor use.

Patients with PH should have a preoperative ECG, chest radiograph, and an echocardiogram to assess ventricular and valvular structure and function. Right-sided heart catheterization is indicated for patients with pulmonary arterial hypertension [78]. The data should be reviewed for characterization of pulmonary hemodynamics (pulmonary artery pressures, pulmonary vascular resistance), cardiac output (looking for signs of right heart failure), and response to vasodilator therapy [79]. Other guidelines for perioperative management include continuing baseline pulmonary vascular targeted therapy (i.e. phosphodiesterase type 5 inhibitors, soluble guanylate cyclase stimulators, endothelin receptor antagonists, and prostanoids) [38]. Preoperative evaluation by a PH specialist can be invaluable in optimizing the patient’s condition prior to surgery and anesthesia.

### Endocrine

#### Diabetes mellitus

Adults with a BMI ≥ 40 are 7 times more likely to have diabetes compared to normal weight individuals [80]. Improved preoperative glycemic control has been associated with decreased postoperative complications and improved diabetes remission rates after bariatric surgery. One retrospective, single center study of 468 patients undergoing bariatric surgery showed that 26.5 % of patients with a Hgb A1c of <6.5 % had a postoperative complication as compared to 36.4 % of patients with a Hgb A1c of > 8 %. Patients with poorly controlled diabetes were more likely to have wound infections, acute renal failure, and postoperative leaks. [81]. The AACE-TOS-ASMBS recommends a target Hgb A1c value of 6.5 % to 7.0 % or less perioperatively, though more liberal targets (Hgb A1c of 7 %-8 %) can be considered in patients with extensive co-morbid conditions or difficult to control diabetes [31].

### Gastrointestinal

#### Liver disease

In parallel with the rising obesity rate, non-alcoholic fatty liver disease (NAFLD) is becoming the most common chronic liver disease worldwide [82]. Histologic changes begin with fatty infiltration (steatosis) and can progress to non-alcoholic steatohepatitis (NASH) once inflammatory changes are superimposed. In 15-20 % of patients, NASH can progress to cirrhosis [83], placing the affected individual at higher risk of developing hepatocellular cancer, portal hypertension, ascites and liver failure [84]. In patients scheduled for weight-loss surgery the prevalence of NAFLD and NASH has been estimated to be as high as 91 % and 37 %, respectively [85, 86]. Risk factors for metabolic syndrome (abdominal obesity, insulin resistance) are also risk factors for NAFLD and NASH [84, 87].

There are currently no serological biomarkers for NAFLD, a commonly asymptomatic condition. NAFLD should be suspected in individuals with metabolic syndrome with or without elevated liver function tests [88]. An elevated triglyceride level may identify obese patients with liver disease. In 160 patients undergoing bariatric surgery, a value >150 mg/dl was associated with a 3.4 fold greater risk of developing NASH. Alanine aminotransferase (ALT) levels > 45 U/l were also associated with NASH. On the other hand, elevated levels of HDL-C decreased the likelihood of NASH [89]. The diagnosis of NAFLD is usually confirmed by imaging studies, most commonly liver ultrasonography. Hernaez *et al.* showed in a meta-analysis that this diagnostic tool had a sensitivity of 84.8 % and specificity of 93.6 % for detecting steatosis affecting greater than or equal to 20-30 % of hepatocytes [90]. Liver biopsy is the gold standard for definitive diagnosis and staging of NAFLD and NASH [91].

NAFLD is not a benign entity. In 229 patients with biopsy-proved NAFLD who were followed for a mean of 26.4 years, Ekstedt *et al.* observed an increase in mortality from cardiovascular and liver-related causes. Notably, advanced fibrosis increased mortality with a hazard ratio of 3.3 (95 % confidence interval 2.27- 4.76) [92]. Though no pharmacologic regimen has been shown to halt progression of liver injury, early diagnosis and intervention with lifestyle modification (e.g. increase in exercise, change in diet), weight reduction and, potentially, participation in clinical treatment trials may help reverse or mitigate hepatic damage [93]. Although NAFLD affects the expression and activity of hepatic enzymes involved in drug metabolism [94], no recommendations currently exist regarding alterations in anesthetic drug dosing in patients with steatosis.

#### Gastroesophageal reflux disease

The prevalence of gastroesophageal reflux disease (GERD) in the USA is reported to range between 18.1 % and 27.8 % [95]. Although gastric emptying is normal in otherwise healthy obese patients [96, 97], mechanical and hormonal changes place obese individuals at higher risk of GERD [98]. Frequent or severe GERD symptoms increase the risk of Barrett’s esophagus [99] and esophageal adenocarcinoma [100].

Infection with *Helicobacter pylori (H. pylori)* is a risk factor for gastroduodenal ulcer disease, gastric cancer and the development of marginal ulcers after gastric bypass surgery [101]. In 611 bariatric surgery patients, 23.7 % had *H. pylori* infection on preoperative endoscopy with biopsy [102]. Proponents of routine preoperative *H. pylori* testing and treatment argue that treatment may decrease postoperative dyspeptic symptoms as well as lower the risk of developing marginal ulcers and gastric cancer [103]. However, routine screening prior to bariatric surgery is not recommended given the paucity of large, controlled trials examining the impact of *H. pylori* on postoperative outcomes [31].

### Hematologic

#### Venous thromboembolism

In addition to the immobilization and venous stasis that characterize the immediate perioperative period, the obesity-related state of chronic inflammation [104] and impaired fibrinolysis [105] place the high BMI patient at an increased risk for postoperative thromboembolic events, a major cause of mortality following bariatric surgery. Compared to a laparoscopic procedure, an open surgical procedure increases the risk of venous thromboembolism (VTE) 4.5 fold [35]. In the LABS-1 study, 0.4 % of 4610 patients undergoing bariatric surgery developed VTE [63]. Among 73,921 patients from the Bariatric Outcomes Longitudinal Database, 0.42 % developed VTE within 90 days of surgery [35]. Furthermore, 73 % of VTE occurred after discharge, with a median interval to VTE event of 14 days. The mortality rate among patients who developed VTE was 25 %. Risk factors for postoperative VTE include increased age, high BMI, male gender, and history of prior VTE.

The American College of Chest Physicians (ACCP) considers obesity to be a high risk factor for VTE [106]. Their guidelines for the prevention of VTE recommend chemoprophylaxis with either low dose unfractionated heparin (LDUH) or low molecular weight heparin (LMWH) in addition to mechanical prophylaxis with elastic stockings or intermittent pneumatic compression. Additionally, the AACE-TOS-ASMBS encourages early ambulation in the postoperative period [31]. Fondaparinux, low dose aspirin, or mechanical prophylaxis is recommended for patients in whom LDUH or LMWH is contraindicated due to heparin allergy or a history of heparin-induced thrombocytopenia [106].

Most venous thromboembolic events occur after discharge [35]. Raftopoulos *et al.* found that 66 % of VTE occurred after cessation of thromboprophylaxis, but continuation of chemoprophylaxis for 10 days post-discharge decreased the risk of VTE in bariatric surgery patients [107]. Accordingly, the AACE-TOS-ASMBS recommends continuing chemoprophylaxis after hospital discharge in high-risk patients (e.g. those with history of DVT) [31].

Inferior vena cava (IVC) filters have been used to prevent VTE in high-risk obese patients. Indications for placement include BMI > 50, history of VTE or venous stasis, and history of hypercoagulable state [108]. Although Birkmeyer *et al.* showed that prophylactic IVC filter placement reduced the rates of postoperative VTE, serious complications, or death/permanent disability [109], the AACP does not recommend IVC filter placement for primary VTE prevention [106].

Obesity-associated alterations to drug volume of distribution and absorption imply that increased doses of LDUH/LMWH may be needed to provide optimum VTE prophylaxis. However, a lack of well-designed and controlled trials precludes specific dosing recommendations. Nevertheless, there are recommendations for therapeutic doses of LMWH. Enoxaparin dosing should be based on total body weight up to a weight of 144 kg [110]. Dalteparin can be dosed based on total body weight up to a weight of 190 kg. In addition, twice daily dosing is recommended because subtherapeutic anti-factor Xa levels are more common with once daily dosing. Monitoring of coagulation in the obese patient can be achieved by measuring anti-factor Xa levels 4 h after a dose.

### Nutrition

Despite excess caloric intake, several nutritional deficiencies have been associated with obesity [111]. Potential contributors include inadequate intake of nutrient-rich food, altered metabolism and bioavailability of micronutrients and coexisting conditions such as secondary hyperparathyroidism. During the pre-bariatric surgery assessment of 267 outpatients with a BMI > 35, Lefebvre *et al.* noticed that many were deficient in 25-hydroxyvitamin D (67.9 %), magnesium (35.4 %), phosphate (21.6 %), iron (18.8 %) and 16.9 % had a low concentration of vitamin A [112]. Micronutrient deficiencies can have important physiologic implications as evidenced by the association between low levels of vitamin D and a higher risk of hypertension, diabetes and cardiovascular diseases [113]. As the postoperative period is generally characterized by changes in food intake and absorption, it is important to address these deficiencies prior to surgery in order to prevent them from worsening afterwards.

## Psychological changes

Obesity is associated with approximately a 25 % increase in mood and anxiety disorders [114]. Data from the National Epidemiologic Survey on Alcohol and Related Conditions (NESARC), a psychiatric epidemiology study of over 40,000 American adults, showed that BMI was significantly associated with most mood and anxiety disorders including depression, mania, panic disorder, social phobia, and personality disorders. Furthermore, there was a 3 to 5 % increased risk of mood and anxiety disorders with every unit increase in BMI [115].

Several causative factors account for the link between obesity and mood disorders. Commonly used atypical antipsychotics, mood stabilizers, and antidepressants often cause some degree of weight gain. Depression is associated with an increase in cortisol levels which is associated with visceral weight gain. Sleep disturbance can result in imbalances in leptin and ghrelin, hormones that mediate appetite and satiety. Depression and obesity are both associated with a low grade state of inflammation as well as a decrease in dopamine activity [116]. Obese individuals also encounter negative societal attitudes resulting in social withdrawal and alienation. One study reported a 12 % prevalence of self-perceived discrimination based on weight, which is comparable to the prevalence of other forms of discrimination such as age and race. Furthermore, as BMI increases so does the perception of bias and discrimination [117].

The presence of mental disorders may complicate post-surgical care. Patients may not have the cognitive capacity to discern the risks and benefits of surgery and may lack the motivation to comply with needed postoperative regimens. Because psychopathology may negatively impact postoperative outcomes, many centers for bariatric surgery require preoperative psychological consultation to ascertain a patient’s mental fitness for undergoing weight loss surgery.

## Other considerations

It is important to keep in mind that obesity is not a single entity but a heterogeneous condition as diverse as cancer: it has many causative factors and affects multiple organ systems resulting in myriad medical and psychological co-morbidities. As such, an all-inclusive medical care plan – including the preoperative preparation – should have a balanced or team-based approach. Comprehensive bariatric surgery programs consist of nurses, nutritionists, internists, psychologists, social workers, physicians and other consultants who work together to ensure the best outcomes at all stages of management. Such an approach is appropriate in the care of the medically complex obese patient presenting for non-bariatric surgery. Improving a patient’s physical condition through a preoperative exercise program, optimization of nutrition and cessation of tobacco and alcohol consumption (“prehabilitation”) can enhance the patient’s functional capacity and, potentially, facilitate post-surgical recovery [118]. Furthermore, severely obese patients should be cared for in high-volume facilities with the specialized expertise, personnel and equipment needed to support all aspects of their perioperative care. Such infrastructure is not cost-effectively maintained in low-volume centers.

## Conclusion

The inflammatory and physiologic changes associated with obesity can result in myriad medical conditions including hypertension, dyslipidemia, T2DM, CAD, stroke, osteoarthritis, OSA, and certain forms of cancers (Table 4). Anesthesiologists can optimize the likelihood of a good outcome by identifying and intervening on factors that might increase the risk of perioperative complications. A thorough medical history to estimate a patient’s functional status along with the cardiac risk associated with the planned procedure should guide preoperative cardiac testing to decrease postoperative MACE. A comprehensive approach to OSA to include routine screening may decrease postoperative respiratory complications. Preoperative management of glucose aiming for Hgb A1c of less than 6.5 % can decrease postoperative surgical complications such as wound infections and leaks. Equally important in the preoperative preparation is the optimization of the patient’s nutritional status and psychological wellbeing. While some data show that we can potentially modify the risk of perioperative complications in the obese patient, much work remains to be done.

## Abbreviations

AACE-TOS-ASMBS: The American Association of Clinical Endocrinologists, the Obesity Society, and the American Society of Metabolic and Bariatric Surgery
ACCP: American College of Chest Physicians
AF: atrial fibrillation
AHI: apnea-hypopnea index
ASA: American Society of Anesthesiologists
biPAP: bilevel positive airway pressure
BMI: body mass index measured in kg/m^2^
CAD: coronary artery disease
CPAP: continuous positive airway pressure
DVT: deep venous thrombosis
ECG: electrocardiogram
GERD: gastroesophageal reflux disease
HDL-C: high-density lipoprotein cholesterol
Hgb A1c: glycosylated hemoglobin
IL-6: interleukin-6
IVC: inferior vena cava
LABS: Longitudinal Assessment of Bariatric Surgery
LDUH: low dose unfractionated heparin
LMWH: low molecular weight heparin
MACE: major adverse cardiac event
MET: metabolic equivalent
NAFLD: non-alcoholic fatty liver disease
NASH: non-alcoholic steatohepatitis
NESARC: National Epidemiologic Survey on Alcohol and Related Conditions
OHS: obesity hypoventilation syndrome
OSA: obstructive sleep apnea
PE: pulmonary embolus
PH: pulmonary hypertension
PSG: polysomnogram
SDB: sleep-disordered breathing
T2DM: type II diabetes mellitus
TNF-α: tumor necrosis factor alpha
USA: United States of America
VTE: venous thromboembolism
